# Extensive molecular screening for hereditary non-polyposis colorectal cancer

**DOI:** 10.1054/bjoc.1999.1014

**Published:** 2000-02

**Authors:** B Dieumegard, S Grandjouan, J-C Sabourin, M-L Le Bihan, I Lefrère, Bellefqih, J-P Pignon, P Rougier, P Lasser, J Bénard, D Couturier, B Bressac-de Paillerets

**Affiliations:** Unité des Marqueurs Génétiques des Cancers, Département d'Anatomie Pathologique, Département de Biostatistiques, Département de Médecine, Département de Chirurgie, Institut Gustave Roussy, 39 rue Camille Desmoulins, Villejuif Cedex, 94805, France; Service d'Hépato-Gastro-Entérologie, Hôpital Cochin, 24 rue du Faubourg Saint-Jacques, Paris, 75014, France

**Keywords:** colorectal cancer, *hMSH2*, *hMLH1*, predisposition, screening

## Abstract

The genetic abnormalities underlying hereditary non-polyposis colorectal cancer (HNPCC) are germline mutations in one of five DNA mismatch repair genes or in the *TGFβRII* gene. The aim of our study was to evaluate the significance of simple tests performed on tumours to select appropriate candidates for germline mutational analysis. We studied three groups of patients, HNPCC kindreds fulfilling the International Collaborative Group (ICG) criteria (*n* = 10), families in which at least one of the criteria was not satisfied (*n* = 7) and sporadic colorectal cancer (CRC) diagnosed before the age of 50 (*n* = 17). We searched for microsatellite instability (MSI), presence of *hMSH2* and *hMLH1* germline mutations, expression of hMSH2, hMLH1 and p53 proteins in tumoural tissue samples by immunostaining. Fifteen out of 17 (88%) of HNPCC and incomplete HNPCC cases were MSI and eight pathogenic germline mutations in *hMSH2* or *hMLH1* were detected in these two groups (53%). All the 17 early-onset sporadic cases were MSS and no germline mutations were detected among the seven investigated cases. Thirteen out of 15 (81%) familial cases were MSI and p53 protein-negative, whereas 13/14 (93%) sporadic cases were MSS and strongly p53 protein-positive. This extensive molecular investigation shows that simple tests such as MS study combined with hMSH2 and hMLH1 protein immunostaining performed on tumoural tissues may provide valuable information to distinguish between familial, and probably hereditary, and sporadic CRC cases. © 2000 Cancer Research Campaign


British Journal of Cancer (2000) 82(4), 871–880
© 2000 Cancer Research Campaign
DOI: 10.1054/ bjoc.1999.1014, available online at http://www.idealibrary.com on IDEAL®

### Extensive molecular screening for hereditary non-polyposis colorectal cancer

**B Dieumegard[1,2], S Grandjouan[2], J-C Sabourin[3], M-L Le Bihan[1], I Lefrère[1,*], Bellefqih[3], J-P Pignon[4], P Rougier[5,†], P Lasser[6], J Bénard[1], D Couturier[2] and B Bressac-de Paillerets[1]**

[1]Unité des Marqueurs Génétiques des Cancers, [3]Département d'Anatomie Pathologique, [4]Département de Biostatistiques, [5]Département de Médecine, [6]Département de Chirurgie, Institut Gustave Roussy, 39 rue Camille Desmoulins, 94805 Villejuif Cedex, France; [2]Service d'Hépato-Gastro-Entérologie, Hôpital Cochin, 24 rue du Faubourg Saint-Jacques, 75014 Paris, France

**Summary** The genetic abnormalities underlying hereditary non-polyposis colorectal cancer (HNPCC) are germline mutations in one of five DNA mismatch repair genes or in the *TGFβRII* gene. The aim of our study was to evaluate the significance of simple tests performed on tumours to select appropriate candidates for germline mutational analysis. We studied three groups of patients, HNPCC kindreds fulfilling the International Collaborative Group (ICG) criteria ($n$ = 10), families in which at least one of the criteria was not satisfied ($n$ = 7) and sporadic colorectal cancer (CRC) diagnosed before the age of 50 ($n$ = 17). We searched for microsatellite instability (MSI), presence of *hMSH2* and *hMLH1* germline mutations, expression of hMSH2, hMLH1 and p53 proteins in tumoural tissue samples by immunostaining. Fifteen out of 17 (88%) of HNPCC and incomplete HNPCC cases were MSI and eight pathogenic germline mutations in *hMSH2* or *hMLH1* were detected in these two groups (53%). All the 17 early-onset sporadic cases were MSS and no germline mutations were detected among the seven investigated cases. Thirteen out of 15 (81%) familial cases were MSI and p53 protein-negative, whereas 13/14 (93%) sporadic cases were MSS and strongly p53 protein-positive. This extensive molecular investigation shows that simple tests such as MS study combined with hMSH2 and hMLH1 protein immunostaining performed on tumoural tissues may provide valuable information to distinguish between familial, and probably hereditary, and sporadic CRC cases. © 2000 Cancer Research Campaign

**Keywords:** colorectal cancer; *hMSH2*; *hMLH1*; predisposition; screening

Colorectal cancer (CRC) is a common malignancy. Estimated new CRC cases rank as the second most common cancer in women and men in France (12). In a subset of cases, an autosomal dominantly inherited susceptibility to CRC has been identified as being responsible for hereditary non-polyposis colorectal cancer (HNPCC) (reviewed in Lynch and Smyrk, 1996). The HNPCC syndrome is characterized by early age of CRC onset, predominance of proximal and multiple tumours (Lynch and Smyrk, 1996). In 1990, the International Collaborative Group (ICG-HNPCC) proposed minimum criteria to be used for selection of families for research collaborative study for the identification of the HNPCC predisposing genes: (i) at least three relatives should have histologically verified CRC and one of them should be a first-degree relative to the other two; (ii) at least two successive generations should be affected; and (iii) in one of the relatives, CRC should be diagnosed under 50 years of age (Vasen et al, 1991). This definition did not include extracolonic cancers, mainly cancers of the endometrium, ovaries, stomach and upper urologic tracts, shown now to be part of the HNPCC tumour spectrum by epidemiological (Lynch et al, 1988; Watson and Lynch, 1993; Benatti et al, 1993; Vasen et al, 1990) and molecular studies (Vasen et al, 1996). Extracolonic cancers have been recently included in new guidelines (Bethesda guidelines) for testing of colorectal tumours for microsatellite instability (Rodriguez-Bigas et al, 1997). The exact frequency of HNPCC is currently unknown; estimates range from 1 to 5% (Lynch and Smyrk, 1996; Aaltonen et al, 1998).

In the past years, germline mutations in six different genes (*hMSH2*, *hMLH1*, *hPMS1*, *hPMS2*, *GTBP*/*hMSH6* and *TGFβRII*) were shown to be responsible for predisposition to colorectal cancers in either HNPCC families (Fishel et al, 1993; Leach et al, 1993; Bronner et al, 1994; Papadopoulos et al, 1994; Nicolaides et al, 1994; Miyaki et al, 1997; Lu et al, 1998), families with incomplete criteria (Moslein et al, 1996; Akiyama et al, 1997; Wang et al, 1997) or patients having developed sporadic CRC before the age of 35 (Liu et al, 1995*a*). Five of these genes encode proteins involved in the DNA repair pathway (Prolla et al, 1994; Fishel et al, 1994; Drummond et al, 1995). Except in rare cases (Nicolaides et al, 1998), the mechanism leading to deficiency in DNA mismatch repair (MMR) is recessive and involves 'two hits': HNPCC patients inherit a mutated allele, then a second somatic genetic or epigenetic event occurs on the wild-type allele during the development of the tumour (Hemminki et al, 1994; Konishi et al, 1996). Through the recognition and repair of incorrectly paired nucleotides, these cancer-susceptibility genes encode cellular

*Received 24 May 1999*
*Revised 11 August 1999*
*Accepted 25 August 1999*

*Correspondence to:* B Bressac-de Paillerets, Unité des Marqueurs Génétiques des Cancers, Institut Gustave Roussy, 39 rue Camille Desmoulins, 94805 Villejuif Cedex, France.

* Present address: Smithkline Beecham Pharmaceutical Laboratories, 4 rue du Chesnay-Beauregard, 35762 Saint Grégoire, France

† Present address: Service d'Hépato-Gastro-Enterologie, Hôpital Ambroise Paré, 9 Avenue Charles de Gaulle, 92104 Boulogne Cedex, France proteins whose function is essential to the integrity of the genome and therefore belong to the 'caretakers' category (Kinzler and Vogelstein, 1997).

HNPCC patients develop CRC at a young age (average age of 44) (Lynch and Smyrk, 1996). Gene-carriers have a lifetime risk of 80% of developing CRC (Vasen et al, 1996) and adapted clinical surveillance should be recommended to maximize detection of early-stage and premalignant lesions in the principal organ sites affected in the HNPCC families (Lynch et al, 1996; Weber, 1996). MMR gene-carriers may also be offered the option of prophylactic subtotal colectomy (Lynch and Smyrk, 1996). Recent data indicate that loss of DNA MMR function might be predictive of tumoural response to chemotherapeutic agents (Aebi et al, 1996). For all these reasons, it is of great clinical importance to accurately identify such high-risk people.

In order to identify such patients, we performed combined molecular approaches among three different groups: HNPCC kindreds fulfilling the ICG criteria ($n$ = 10), families in which at least one of the criteria was not satisfied ($n$ = 7) and apparently sporadic CRC cases diagnosed before the age of 50 ($n$ = 17). Our results suggest that MSI and absence of p53 protein accumulation in tumour are predictive of familial and probably hereditary CRC cases. In addition, the study of MS status combined with hMSH2 and hMLH1 protein immunostaining performed on tumoural tissues represents a low-cost discriminating test for selecting CRC cases with the highest probability to have an MMR-gene germline defect.

### MATERIALS AND METHODS

### Family and patient selection

Thirty-four white patients with CRC were selected for the study, with their written informed consent. Group 1 ($n$ = 10) represented patients fulfilling the ICG criteria for HNPCC. In group 2 ($n$ = 7), patients were classified as incomplete HNPCC, because they had a strong family history of CRC, but were missing at least one ICG criterion. Group 3 ($n$ = 17) represented a clinic-based population prospectively selected solely upon the criterion of age being below 50 years at diagnosis of the CRC (range 17–50, mean 36), after verification of the absence of any CRC and tumours from the HNPCC spectrum recorded in their families up to the second degree. All cancers were histologically verified.

### Tissue samples and DNA preparation

Frozen or paraffin-embedded tissues were examined microscopically for determination of exact tumour and normal mucosa localization. DNA was prepared from tumoural tissue samples after dissection upon the histological analysis. Germinal DNA was obtained from peripheral blood mononuclear cells drawn at the same time that cancer tissue was obtained.

### Analysis of microsatellite instability

Paired normal and tumoural DNA was analysed for each patient for microsatellite instability with a set of 20 microsatellite markers, dinucleotide (CA)n repeats: D2S116, D2S117, D2S119, D2S123, D2S147, D2S155, D2S391, D3S1277, D3S1298, D3S1561, D5S82, D5S299, CA7, D5S346, D7S481, D7S517, D7S531, D11S904, D13S175, D20S116 (Gyapay et al, 1994). In addition, three mononucleotide (A)n repeats were investigated as previously described, BAT-40 (Liu et al, 1996), BAT-26 (Parsons et al, 1995) and BAT-RII (Parsons et al, 1995). Polymerase chain reaction (PCR) reaction mix (20 µl) contained 100 ng of DNA (extracted from lymphocytes or tumour), 0.15 µM of each primer, 5 µM of each dNTP, [$^{\alpha\text{-}33}$P]-dATP, 1.25 mM magnesium chloride ($MgCl_2$), 1 × PCR buffer, 0.5 U *Taq* Polymerase (Perkin-Elmer). PCR was performed as follows: one initial denaturing step at 95°C for 5 min; 30 cycles at 95°C (30 s), 55°C (30 s) and 72°C (30 s); a final extension step at 72°C for 10 min. PCR products were diluted with formamide dye solution, followed by denaturation at 95°C (2 min). Four microlitres of each diluted sample were loaded on 8% polyacrylamide and 7-M urea gels. After electrophoresis, dried gels were exposed to X-ray films at room temperature. Tumours were scored according to the number of unstable mononucleotide and dinucleotide microsatellites, and MS status was defined as MSS for tumour displaying less than 10% of unstable loci and MSI for tumour displaying more than 10% of unstable loci.

### DNA analysis of *hMSH2* and *hMLH1* genes

Genomic DNA was isolated from blood samples or lymphocytes. DNA was PCR-amplified for single-strand conformation polymorphism (SSCP) analysis of *hMSH2* and *hMLH1* coding sequences. The reaction mixture (20 µl) contained 100 ng of genomic DNA, 0.15 µM of each primer (forward and reverse primers were located in the sequences of the intron–exon boundaries of the *hMSH2* and *hMLH1* genes, kindly provided by B Vogelstein, Johns Hopkins University, and R Kolodner, Dana-Farber Cancer Institute; primers are available upon request), 5 µM of each dNTP, [$^{\alpha 33}$P]-dATP, 1.25 mM $MgCl_2$, 1 × PCR buffer and 0.5 U *Taq* Polymerase (Perkin-Elmer). PCR was performed as follows: one initial denaturing step at 95°C for 5 min; 35 cycles at 95°C (1 min), 55°C (1 min) and 72°C (1 min); a final extension step at 72°C for 10 min. PCR products were diluted tenfold with formamide dye solution, followed by denaturation at 95°C (2 min). SSCP analysis was performed as previously described (Soufir et al, 1998). Aberrant single-strand DNA fragment samples were analysed by direct sequencing. PCR products were purified with Microspin columns S-400HR (Pharmacia) and sequenced with the Thermosequenase kit (Amersham), following manufacturer's instructions. Primers for sequencing were the same as those used for PCR-SSCP.

Total RNA was isolated from lymphocytes or lymphoblastoid cell lines using RNA-B™ (Bioprobe) according to the manufacturer's instructions. cDNAs were reverse transcribed as previously described (Soufir et al, 1998). One microlitre of cDNA templates was PCR-amplified in a final volume of 50 µl as follows: 7.5 pmol of each primer, 200 µM of each dNTP (Pharmacia), 1.5 mM $MgCl_2$ and 1.25 U *Taq* Gold Polymerase (Perkin-Elmer). Primers are available upon request. The PCR products were submitted to electrophoresis on agarose gel and visualized by ethidium bromide staining to check for the presence of abnormal bands.

For six cases, somatic allelic losses were assessed by comparing sequencing data of matched lymphocytes and tumoural-extracted DNA at the site of the germline missense *hMLH1* mutations. Experiments were performed four times using either dye primers or dye dideoxynucleotides sequencing kits on both strands and sequencing patterns were reproducible.

 *© 2000 Cancer Research Campaign*

### Immunohistochemical analysis

Immunoperoxidase staining for hMLH1, hMSH2 and p53 proteins in formalin-fixed, paraffin-embedded or frozen tissue sections, was performed by a streptavidin–biotin diaminobenzidine technique as previously described (Gompel et al, 1994). Briefly, slides were incubated overnight at 4°C with mouse monoclonal antibodies to hMLH1 (clone Ab-1, 10 μg $ml^{-1}$, Oncogene Research Products) or hMSH2 (clone FE11, 5 μg $ml^{-1}$, Oncogene Research Products) or p53 (clone DO7, 2 μg $ml^{-1}$, Dako). For frozen sections, immunohistochemical analysis was performed with antibodies at 5 μg $ml^{-1}$ for hMLH1 and 2 μg $ml^{-1}$ for hMSH2, 1 μg $ml^{-1}$ for p53. Normal tissue adjacent to tumour was used as internal positive control. The normal staining pattern for both hMLH1 and hMSH2 was nuclear. Tumour cells that exhibited an absence of nuclear staining in the presence of non-neoplastic cells with nuclear staining were considered to have an abnormal pattern. Inversely, normal cells displayed no p53 staining, whereas nuclear staining was a feature of tumoural samples.

### RESULTS

### Clinical features of CRC patients

In our three groups, cancer site and age at diagnosis were recorded for each patient, as well as family history of CRC and cancers at other sites (Table 1).

### Microsatellite instability

First, we looked for microsatellite instability in tumours developed by patients. Fifteen cases were stated as MSI and 19 cases were stated as MSS (Table 2). Two cases (CHO and SCA) displayed

**Table 1** Clinical features of CRC patients

| Patient identity | Cancer site and age at diagnosis[b,c] | CRC in the family[d] | Cancers at other sites in the family |
|---|---|---|---|
| **HNPCC** | | | |
| AUB | R* (47) | 3 | Lung |
| HAL | L (45); E* (48) | 3 | E |
| JEN | R* (42); L (43) | 4 | E |
| GRI | R* (26) | 4 | E |
| DUV | L* (29) | 5 | Ovary |
| BLA | E (54); R* (55); Bl (55); Ba (47;57) | 3 | E, breast |
| BES | R* (55), L (55) | 3 | 0 |
| CHO | R, L, Re* (31) | 3 | 0 |
| BIL | Re (36), R* (42) | 4 | Lung, Ke, Sp |
| RIE | R* (35) | 3 | E |
| **Incomplete HNPCC[a]** | | | |
| BAR | L* (52) | 3 | Breast |
| FAR | L* (48) | 2 | Pancreas |
| PLO | T* (37) | 2 | E |
| CHI | L (57); R* (66) | 2 | St, sarcoma |
| MAL | E (48); Re (56); R (63); L (65); T* (67) | 2 | E, breast, Ba |
| MER | R* (52), E (52) | ? | ? |
| SCA | Re* (50); St (50) | 2 | Lung, gynaecological |
| **Sporadic CRC** | | | |
| DAC | R* (17) | 0 | 0 |
| FRO | Re* (20) | 0 | Lung, breast |
| DEL | L (25); Re* (26) | 0 | 0 |
| DON | T (28); L* (31) | 0 | 0 |
| GAN | Re* (32) | 0 | 0 |
| VIT | R* (34) | 0 | Prostate, brain |
| COU | L* (42) | 0 | Breast |
| MEN | L* (35) | 0 | E, prostate, ORL |
| HUC | Re* (36) | 0 | Breast |
| CHA | L* (37) | 0 | Breast, lung, testis |
| SKO | L* (38) | 0 | 0 |
| CAM | L* (41) | 0 | 0 |
| PER | L* (42) | 0 | Lung |
| BEL | L* (44) | 0 | 0 |
| LAM | T* (44) | 0 | 0 |
| DES | L* (47) | 0 | 0 |
| DAS | Re* (50) | 0 | ? |

[a] Missing ICG-HNPCC criterion: (i) only two relatives have colorectal cancer (FAR, PLO, CHI, MAL, SCA); (ii) in one of the relatives, colorectal cancer was diagnosed after 50 years of age (BAR). Abbreviations are R: ascendant colon; L: descendant colon and sigmoid; T: transverse colon; E: endometrium; Re: rectum; St: stomach; Ke: keratoacanthoma; Sp: spino-cell carcinoma; Ba: basal cell carcinoma. [c] Tissue studied for MS analysis and immunostaining are noted *. [d] Up to the second degree for sporadic cases.

© 2000 Cancer Research Campaign 

**Table 2** Microsatellite markers analysis, MMR status (genes and proteins) and p53 protein alterations

| Patient | Total MSI | MS status | Gene[b] | hMSH2 protein[c] | hMLH1 protein[c] | p53 status[c,d] |
|---|---|---|---|---|---|---|
| **HNPCC** | | | | | | |
| AUB | 16/18 (89%) | + | hMLH1? | NT | NT | NT |
| HAL | 0/21 (0%) | – | None | + | + | – |
| JEN | 7/7 (100%) | + | None | + | + | + |
| GRI | 12/17 (70%) | + | None | + | – | – |
| DUV | 6/7 (86%) | + | hMLH1 | + | – | – |
| BLA | 6/7 (86%) | + | hMSH2 | – | + | – |
| BES | 16/23 (70%) | + | hMLH1 | + | + | – |
| CHO | 5/18 (28%) | +[a] | None | – | + | – |
| BIL | 12/15 (80%) | + | hMSH2 | + | + | – |
| RIE | 3/5 (60%) | + | hMLH1 | NT | NT | – |
| **Incomplete HNPCC** | | | | | | |
| BAR | 1/23 (4%) | – | None | + | + | + |
| FAR | 12/15 (80%) | + | None | + | – | – |
| PLO | 21/23 (91%) | + | hMSH2 | – | + | – |
| CHI | 10/17 (59%) | + | hMLH1 | + | – | – |
| MAL | 6/7 (86%) | + | hMLH1 | + | – | – |
| MER | 18/23 (78%) | + | None | + | – | – |
| SCA | 4/23 (17%) | +[a] | None | + | – | – |
| **Sporadic CRC** | | | | | | |
| DAC | 0/23 (0%) | – | None | + | + | + |
| FRO | 0/10 (0%) | – | None | + | + | + |
| DEL | 0/23 (0%) | – | None | + | + | + |
| DON | 0/23 (0%) | – | None | + | + | + |
| GAN | 0/20 (0%) | – | None | + | + | + |
| VIT | 1/24 (4%) | – | None | + | + | – |
| COU | 1/20 (5%) | – | None | + | + | + |
| MEN | 0/23 (0%) | – | NT | + | NT | NT |
| HUC | 1/24 (4%) | – | NT | ND | ND | + |
| CHA | 0/23 (0%) | – | NT | + | + | NT |
| SKO | 0/23 (0%) | – | NT | + | + | NT |
| CAM | 0/22 (0%) | – | NT | + | + | + |
| PER | 0/23 (0%) | – | NT | + | + | + |
| BEL | 0/23 (0%) | – | NT | + | + | + |
| LAM | 0/19 (0%) | – | NT | + | + | + |
| DES | 0/21 (0%) | – | NT | + | + | + |
| DAS | 0/23 (0%) | – | NT | + | + | + |

[a] These two cases were classified as MSI because they presented a clear instability of mononucleotide (A)n repeats (3/3 and 2/3, respectively) whereas none of the 19 MSS cases displayed any instability of the 3 mononucleotide (A)n repeats tested (data not shown). [b] See details of germline mutations on Table 3. [c] NT: not tested; ND: not determined, in relation with data of insufficient quality. [d] p53 status: negative, <10% positive nuclear staining cells; positive > 40% positive nuclear staining cells.

MSI values of 28% and 17% respectively, below the 20–30% cut-off used usually to define MSI (Bocker et al, 1997; Dietmaier et al, 1997). We classified these two cases as MSI because they presented clear instability of mononucleotide (A)n repeats (3/3 and 2/3 respectively) whereas none of the 19 MSS cases displayed any instability of the three mononucleotide (A)n repeats tested (data not shown).

Analysis of MS status according to patient's group showed a clear-cut distribution, 15 out of 17 (88%) of HNPCC and incomplete HNPCC cases were MSI, whereas all 17 early-onset sporadic cases (100%) were MSS (Table 2). The sensitivity of MSI detection is 88% (95% confidence interval (CI) 56–99%) and its specificity is 100% (80–100%).

### Germline mutation of *hMSH2* and *hMLH1* genes

We screened for *hMSH2* and *hMLH1* germline mutations in all 15 MSI cases as well as in nine MSS cases, including seven cases selected among the early-onset sporadic group. We detected five non-ambiguous germline mutations (frame-shift or splice site) in HNPCC or incomplete HNPCC cases (Tables 2 and 3). In addition, we detected five mis-sense mutations in *hMLH1* gene and one in *hMSH2* gene. Screening for abnormally-sized cDNA allowed us to detect deletion of exon 13 in *hMSH2* gene from patients BIL and COU which correspond to an alternative transcript (Xia et al, 1996; Mori et al, 1997) and deletion of exon 17 of *hMLH1* gene from patients CHI and SCA. In the case of CHI, loss of exon 17 may be explained by the existence of a genomic mis-sense mutation, Glu663Gly, this codon being the last one of exon 17. In the SCA case, no underlying genomic mutation was detected by our screening methods. This exon skipping may be non-pathogenic as an exon 17 splice variant was first reported in a patient displaying an *hMLH1* exon 15 splice donor site mutation (Kohonen-Corish et al, 1996) and subsequently in 15/15 PBMC samples (Genuardi et al, 1998*b*). We observed loss of hMLH1 expression in the tumour developed by patient SCA (Table 3), which can be the consequence of *hMLH1* gene somatic inactivation by mutation or hypermethylation (Thibodeau et al, 1996; Kane et al, 1997). We also performed an SSCP screening for all

 © 2000 Cancer Research Campaign

**Table 3** Validation of hMSH2 and hMLH1 germline mutations

| Patient | Gene | Exon | Codon | Nucleotide change[a] | Consequence[b] | Tumoural LOH | Segregation | Controls[c] | Published mutation[d] | Loss of function[e] | Conclusion |
|---|---|---|---|---|---|---|---|---|---|---|---|
| AUB | hMLH1 | 19 | 716 | G2146A | Val 716 Met | Loss WT | Yes, 2 cases | 0/192 | Yes[1] | NT | Uncertain significance |
| DUV | hMLH1 | Intron 6 | – | A546-2G | Deletion exon 7 | NT | Yes, 5 cases | NT | Yes[1] | NT | Mutation |
| BLA | hMSH2 | 5 | 280 | 840insT | Frame-shift | NT | Yes, 2 cases | NT | Yes[2] | NT | Mutation |
| BES | hMLH1 | 4 | 117 | C350T | Thr 117 Met | Loss WT | NT | 0/192 | Yes[1] | Yes | Mutation |
| CHO | hMLH1 | 19 | 718 | C2152T | His 718 Tyr | none | NT | 0/192 | Yes[1] | NT | Likely polymorphism |
| BIL | hMSH2 | 14 | 751 | G2251A | Gly 751 Arg | NT | Yes, 3 cases | 0/192 | No | NT | Likely mutation |
| RIE | hMLH1 | 11 | 332 | 994delA | Frame-shift | NT | Yes, 3 cases | NT | No | NT | Mutation |
| PLO | hMSH2 | 15 | 828 | dup 14bp2484 | Frame-shift | NT | Yes, 2 cases | NT | No | NT | Mutation |
| CHI | hMLH1 | 17 | 663 | A1988G | Glu663Gly/deletion exon 17 | Loss WT | NT | 0/192 | No | NT | Mutation |
| MAL | hMLH1 | Intron 14 | – | C1668-3A | NT | Loss WT | NT | NT | No | NT | Mutation |
| SCA | hMLH1 | | | NI | Deletion exon 17 | NT | NT | NT | No | NT | Splice variant |
| DAC | hMLH1 | 11 | 326 | T977C | Val 326 Ala | none | NT | 0/192 | Yes[1] | No | Polymorphism |

[a] NI: not identified by the available techniques. [b] NT: not tested. [c] Allele frequency in control chromosomes from 96 unrelated individuals. [d] [1]Mutation reported in the ICG-HNPCC database (http://www.nfdht.nl) and/or the literature; [2]mutation already published by the authors. [e] Reference: Shimodaira et al (1998).

mis-sense mutations in a control population of 96 unrelated individuals belonging to families with inherited predisposition to breast/ovarian cancers, melanoma or polyposis except HNPCC families. All samples displaying abnormal migration pattern were used as positive SSCP controls. Results are indicated in Table 3. None of the mis-sense mutations were detected in the 96 unrelated controls. For family BIL, we were able to perform segregation analysis and we detected hMSH2 Gly751Arg germline mutation in 3/3 affected members and not in five unaffected members (data not shown). In the four other cases, segregation analysis was not possible. In 14 patients, neither *hMSH2* nor *hMLH1* germline mutations were detected, nine were MSS and five were MSI cases. These negative results are likely to be underestimated because we did not search for either gross genetic abnormalities or inferred regulatory mutations in *hMSH2* and *hMLH1* genes, or for any kind of alterations in *PMS1*, *hPMS2*, *hMSH6/GTBP* and *TGFβRII* genes.

### Tumoural LOH for validation of mis-sense mutations

Overall, we detected six germline missense mutations and in the absence of a functional test, we had difficulties in discriminating between variants and disease-causing mutations for five of them. Except in rare cases of dominant mutations, deleterious mis-sense mutations in tumour suppressor genes are followed by a second somatic event acquired in tumoural cells such as allelic loss, point mutation or hypermethylation. In order to detect tumoural loss of heterozygosity (LOH), we performed automated sequencing of DNA extracted from microdissected tumours at the site of five germline mis-sense mutations using a positive control with unambiguous mutation (MAL patient). Four cases (MAL, CHI, AUB, BES) showed a decrease of wild-type allele in tumoural DNA as compared to lymphocyte-extracted DNA, whereas two cases (CHO and DAC) had identical sequencing patterns in lymphocyte and tumour-extracted DNA (Figure 1). These results showing loss of *hMLH1* wild-type allele in three tumours in addition to the positive control, argue for a pathogenetic role for Glu663Gly, Val716Met and Thr117Met mutations. In addition, Thr117Met scored as a loss-of-function mutation in a functional assay (Shimodaira et al, 1998). The significance of *hMLH1* Val716Met germline mis-sense mutation still remains uncertain because it was detected in two patients with MSS tumour (Genuardi et al, 1998*a*; S Olschwang, personal communication). This mutation could be non-pathogenic and the *hMLH1* tumoural LOH that was observed could be related to unidentified *hMLH1* germline or somatic events. No tumoural LOH was seen for Val326Ala and His718Tyr mutations. Kowalski et al found an allele frequency of 0.14 for His718Tyr among an Afro-American population (Kowalski et al, 1997), suggesting that this mutation represents a polymorphism. *hMLH1* Val326Ala did not score as a loss-of-function mutation in a functional assay and therefore is likely to be a rare variant (Shimodaira et al, 1998). Overall, in the absence of a functional test and segregation analysis (no living affected relatives exist in severely affected HNPCC families), the analysis of second somatic events in the tumoural DNA is valuable in order to classify mis-sense mutations as simple variants or disease-causing mutations.

### Correlation of hMSH2 and hMLH1 protein expression with MS status and germline mutations

Protein expressions were examined in patients' tumours. Lack of hMLH1 or hMSH2 expression was observed in ten tumours, all of them being MSI and removed from family cases (HNPCC or incomplete HNPCC) (Table 2 and Figure 2). No cases had concomitant loss of hMSH2 and hMLH1. All sporadic MSS tumours tested (15 cases) displayed normal staining pattern for hMLH1 and hMSH2. The absence of one of the two MMR gene products was associated with the presence of a validated germline mutation in the corresponding gene in five cases (DUV, BLA,

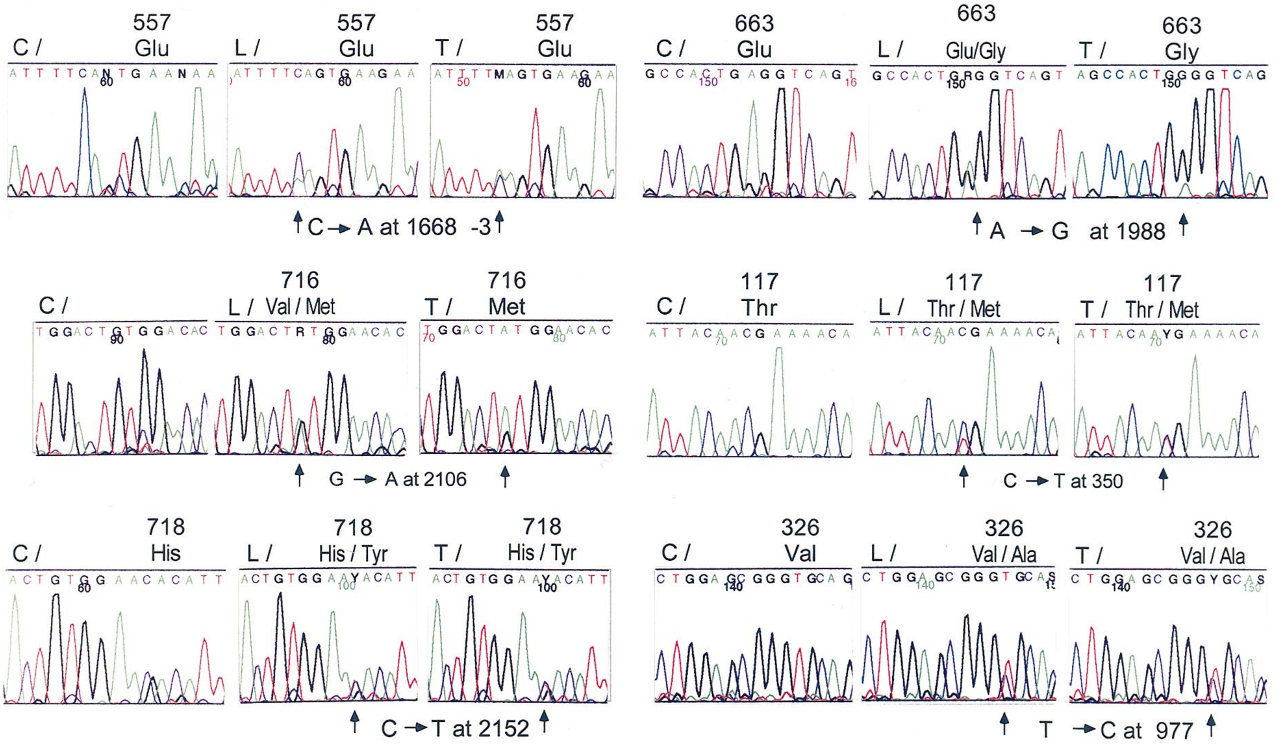

**Figure 1** Analysis of tumoral LOH at germinal mutation sites for six cases. For each patient, three sequencing patterns are shown, a wild-type control (C), patient's lymphocytes (L) and tumour (T). Germline mutations are described, codons (above) and arrows point to the exact mutated nucleotides (beneath). From upper left to lower right: four cases (MAL, CHI, AUB, BES) show a decrease of wild-type allele in tumoural DNA, whereas two cases (CHO and DAC) have identical sequencing patterns in lymphocytes and tumour-extracted DNA

PLO, CHI and MAL), but not in four cases without detected germline mutation. In these four cases, the observed loss of hMSH2 or hMLH1 expression could be related to inferred regulatory germline mutation, somatic mutation and in addition, for *hMLH1* to promoter hypermethylation (Herman et al, 1998). It should be noticed that cases BES and BIL have validated *hMSH2* or *hMLH1* mis-sense germline mutations (Glu751Arg and Thr117Met respectively) and normal hMSH2 and hMLH1 expression in their tumour. As previously stated, mis-sense mutations might give rise to normal levels of a non-functional protein (Thibodeau et al, 1996). Indeed, the hMSH2-deficient ovarian tumour cell line 2774, despite the presence of an Arg524Pro mis-sense mutation in exon 14 of *hMSH2* gene, produces a full-length mutant protein (Orth et al, 1994; Richards et al, 1997).

### Correlation of p53 protein expression with MS status and hMSH2/hMLH1 germline mutations

We also explored p53 status in our three groups of patients. There is a good correlation between *p53* gene mutations and p53 protein accumulation at a cellular level (Casey et al, 1996), therefore we examined p53 protein expression in all available samples. In some cases, tumoural tissues were totally consumed by previous experiments and p53 status could not be determined. Independently to molecular results, p53 staining was first scored by a pathologist in four categories: –, no staining; +, 0–10% positive cells; ++, 10–50% positive cells; +++, 50–100% positive cells (data not shown). At a second examination, it was noticed that all + cases were 0–5% positive cells, whereas ++ cases were 40–50% positive cells. Therefore, a final p53 status was given, negative for tumours displaying less than 5% nuclear p53 staining cells and positive for tumours displaying more than 40% nuclear p53 staining cells (Table 2). Negative p53 staining was detected in all family cases tumours, mainly MSI, except two (JEN and BAR); positive p53 staining was detected in all sporadic cases which are also MSS tumours, except one (VIT). Examples are shown in Figure 2. The proportion of MSI and p53 negative staining was 81% (13/16) among family cases (HNPCC and incomplete HNPCC) and 0% (0/14) among sporadic CRC (Fisher's exact test, $P < 0.00001$).

### DISCUSSION

In order to identify hereditary CRC, we performed combined molecular approaches among three different groups, HNPCC kindreds fulfilling the ICG criteria, families in which at least one of the criteria was not satisfied and apparently sporadic CRC cases diagnosed before the age of 50.

Analysis of MS status in relation with patient's groups showed a clear-cut distribution, 15 out of 17 (88%) of HNPCC and incomplete HNPCC cases were MSI, whereas all 17 early-onset sporadic cases were MSS. This observation of absence of MSS sporadic tumours in our series is not in accordance with other reported studies. The 17 sporadic cases with early onset were selected prospectively upon precise criteria (age being below 50 years at diagnosis of CRC; absence of any CRC and tumours from the HNPCC spectrum recorded in their families up to the second degree) in a clinic-based population. MMR deficiency based on an MSI status has been reported in sporadic CRC at varying

 *© 2000 Cancer Research Campaign*

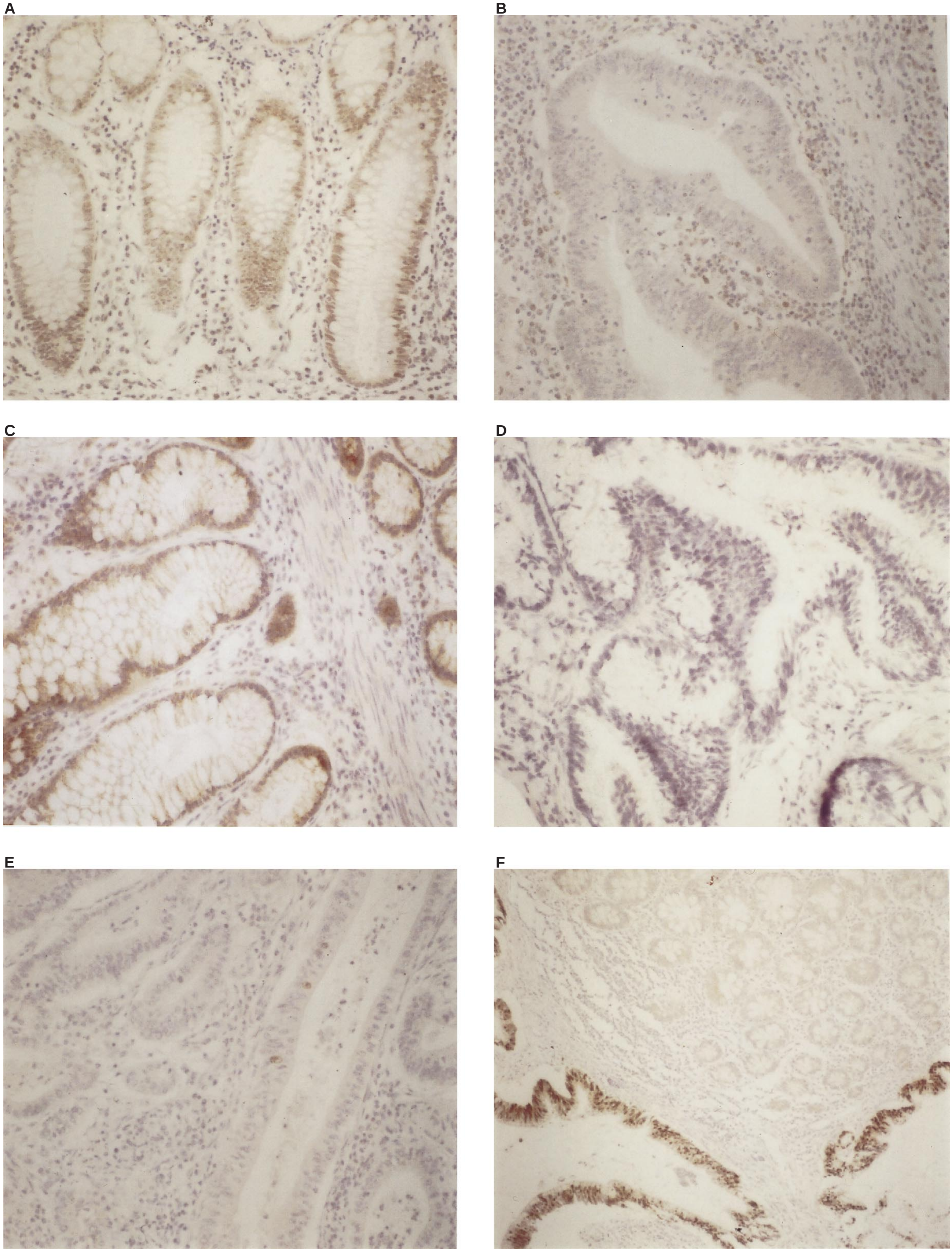

**Figure 2** Immunohistochemical detection of hMSH2 in normal colonic epithelium (**A**) and absence of staining of epithelial neoplastic cells (**B**) for patient BLA; immunohistochemical detection of hMLH1 in normal colonic epithelium (**C**) and absence of staining of epithelial neoplastic cells (**D**) for patient GRI; absence of p53 staining in an MSI adenocarcinoma, patient BLA (**E**) and positive staining in an MSS adenocarcinoma, patient DON (**F**)

*© 2000 Cancer Research Campaign*

frequency in several studies: 13% of 273 samples (compiled data in Kinzler and Vogelstein, 1996), 36% of 77 samples (Liu et al, 1995*a*) and 15% of 88 samples (Olschwang et al, 1997). In these earlier studies, selection against family history was less stringent than in our study, in which only CRC cases from HNPCC families according to ICG criteria were excluded. Therefore, an unknown fraction of these tumours could have been stated as incomplete HNPCC according to our criteria and might represent false sporadic cases. Additionally, sporadic CRC cases might have been selected upon additional criteria, such as CRC location, among a large population of sporadic CRC. Indeed, in another study describing 19 sporadic MSI CRC, some of which displayed somatic *hMSH2* mutations, 18 were right-sided (Borresen et al, 1995). In our series of 17 sporadic CRC cases only two tumours were right-sided. Therefore, tumour location in colon may partly explain differences between our results and previously reported frequent sporadic MSI CRC. Moreover, a model explaining the relationship between MMR genes dysfunction and tumour location in right colon has been recently proposed (Janin, 1999).

The absence of MSI among 17 early-onset sporadic cases indicates that MMR genes inactivation, either germline or somatic, is a rare event in sporadic CRC. Concerning germline mutations, a 6% (3/50) prevalence of *hMSH2* and *hMLH1* germline mutations was reported in a cohort of CRC cases diagnosed before the age of 45 and without a family history of any HNPCC cancers (Tomlinson et al, 1997). This prevalence may have been overestimated because the three germline mutations detected were mis-sense, one at least being a known polymorphism (hMLH1, Ile219Val) (Liu et al, 1995*b*). Therefore, genetic susceptibility other than MMR gene germline mutations and/or environmental factors are responsible for the majority of CRC developed by young patients (reviewed in Burt, 1996). In addition, although our series is small and some MMR gene somatic mutations have been reported (Borresen et al, 1995; Thibodeau et al, 1996), our results (i.e. 17 sporadic tumours displaying MSS and hMSH2/hMLH1 protein immunostaining) indicate that somatic inactivation of *hMSH2* and *hMLH1* genes is not a frequent event in sporadic colorectal carcinogenesis, at least for patients younger than 50. Therefore, colorectal tumorigenesis may represent a second example of the existence of two different genetic pathways for sporadic and inherited cancers, after the breast and ovarian cancer model (Futreal et al, 1994; Merajver et al, 1995).

The second point addressed in this work was to explore p53 status in relation with the MMR pathway status. We examined p53 protein expression in all available tumoural tissue samples by immunohistochemistry. Negative p53 staining was detected in all family cases tumours, mainly MSI, except two, and positive p53 staining was detected in all sporadic and MSS tumours except one. Similar correlation has recently been reported (Fujiwara et al, 1998). *p53* gene somatically-acquired mutations occur very frequently during the development of CRC (Baker et al, 1989). Such mutations lead to p53 protein accumulation probably in relation with loss of mdm2-mediated down-regulation (Midgley and Lane, 1997). Absence of p53 staining in MSI tumours could be related to (i) accumulation of multiple p53 mutations leading to the absence of p53 protein and (ii) wild-type p53 protein. Several studies reporting low incidence of *p53* mutation in MSI tumours imply that the second hypothesis may be relevant for the majority of MSI/p53- cases (Ionov et al, 1993; Losi et al, 1997; Olschwang et al, 1997). The significant correlation that we observed between microsatellite status and p53 protein accumulation may have a molecular explanation. Two alternative genetic pathways have been described in colorectal carcinogenesis, with either APC gene or MMR gene inactivation (Olschwang et al, 1997). In the APC pathway, the defective APC protein is unable to promote the proteolytic degradation of β-catenin, leading to its accumulation (Munemitsu et al, 1995) and it has been recently reported that overexpression of β-catenin results in accumulation of p53, possibly through interference with its proteolytic degradation (Damalas et al, 1999). Therefore, the correlation between MSS (absence of MMR gene inactivation and probable APC gene inactivation) and p53 protein accumulation, makes sense.

To summarize, 13 out of 16 (81%) family cases were MSI and p53 protein-negative, whereas 13/14 (93%) sporadic cases were MSS and p53 protein-positive. Such observations support the concept of the existence of two different genetic pathways in colorectal carcinogenesis (Olschwang et al, 1997). The first pathway exists mainly in family cases and is characterized by MMR gene inactivation without p53 protein accumulation. The second, which is more frequent in sporadic cases, is characterized by p53 protein accumulation without MMR gene inactivation. Therefore, simple tests performed on tumoural tissues, such as MS study combined with hMSH2 and hMLH1 protein immunostaining, provide valuable information to select cases relevant to germline mutation screening.

### ACKNOWLEDGEMENTS

We would like to thank Drs MF Avril, D Cattan, S Chaussade, JJ Delpierre, M Ducreux, MC Fabri, M Gibon, R Guimbaud, Y Le Mercier, A Louvel, M Mahé, D Mimram, E Pines for clinical management and sample collection; Drs Caroline Bognel and Pierre Duvillard for advices; Drs Sylviane Olschwang and Thierry Soussi for useful discussion; Drs Nicolas Janin and Alain Puisieux for critical reading of the manuscript; Josyane Le Calvez and Johny Bombled for technical assistance. This work was supported by grants from Institut Gustave Roussy (CRC N°95–16) and from the Ligue des Pyrénées Orientales. BD was a 'Médaille de l'internat' recipient and was supported by the 'Fonds d'Etude de Recherche des Hôpitaux, AP-HP'; SB was supported by the Ligue Nationale contre le Cancer.

### REFERENCES

Aaltonen LA, Salovaara R, Kristo P, Canzian F, Hemminki A, Peltomaki P, Chadwick RB, Kaariainen H, Eskelinen M, Jarvinen H, Mecklin JP and de la Chapelle A (1998) Incidence of hereditary nonpolyposis colorectal cancer and the feasibility of molecular screening for the disease. *N Engl J Med* **338:** 1481–1487

Aebi S, Kurdi-Haidar B, Gordon R, Cenni B, Zheng H, Fink D, Christen RD, Boland CR, Koi M, Fishel R and Howell SB (1996) Loss of DNA mismatch repair in acquired resistance to cisplatin. *Cancer Res* **56**: 3087–3090

Akiyama Y, Sato H, Yamada T, Nagasaki H, Tsuchiya A, Abe R and Yuasa Y (1997) Germ-line mutation of the *hMSH6/GTBP* gene in an atypical hereditary nonpolyposis colorectal cancer kindred. *Cancer Res* **57**: 3920–3923

Baker SJ, Fearon ER, Nigro JM, Hamilton SR, Preisinger AC, Milburn Jessup J, van Tuinen P, Ledbetter DH, Barker DF, Nakamura Y, White R and Vogelstein B (1989) Chromosome 17 deletions and p53 gene mutations in colorectal cancer. *Science* **244**: 217–221

Benatti P, Sassatelli R, Roncucci L, Pedroni M, Fante R, Digregorio C, Losi L, Gelmini R and Deleon MP (1993) Tumour spectrum in hereditary non-polyposis colorectal cancer (HNPCC) and in families with 'suspected HNPCC' – a population-based study in northern Italy. *Int J Cancer* **54**: 371–377

*© 2000 Cancer Research Campaign*

Bocker T, Diermann J, Friedl W, Gebert J, Holinski-Feder E, Karner-Hanush J, von Knebel-Doeberitz M, Koelble K, Moeslein G, Schackert H, Wirtz H, Fishel R and Rüschoff J (1997) Microsatellite instability analysis: a multicenter study for reliability and quality control. *Cancer Res* **57**: 4739–4743

Borresen A, Lothe RA, Meling GI, Lystad S, Morrison P, Lipford J, Kane MF, Rognum TO and Kolodner RD (1995) Somatic mutations in the hMSH2 gene in microsatellite unstable colorectal carcinomas. *Hum Mol Genet* **4**: 2065–2072

Bronner CE, Baker SM, Morrison PT, Warren G, Smith LG, Lescoe MK, Kane M, Earabino C, Lipford J, Lindblom A, Tannergard P, Bollag RJ, Godwin AR, Ward PC, Nordenskjold M, Fishel R, Kolodner R and Liskay RM (1994) Mutation in the DNA mismatch repair gene homologue *hMLH1* is associated with hereditary non-polyposis colon cancer. *Nature* **368**: 258–261

Burt RW (1996) Familial risk and colon cancer. *Int J Cancer (Pred Oncol)* **69**: 44–46

Casey G, Lopez ME, Ramos JC, Plummer SJ, Arboleda MJ, Shaughnessy M, Karlan B and Slamon DJ (1996) DNA sequence analysis of exons 2 through 11 and immunohistochemical staining are required to detect all known p53 alterations in human malignancies. *Oncogene* **13**: 1971–1981

Damalas A, Ben-Ze'ev A, Simcha I, Shtutman M, Leal JF, Zhurinsky J, Geiger B and Oren M (1999) Excess beta-catenin promotes accumulation of transcriptionally active p53. *EMBO J* **18**: 3054–3063

de Vathaire F, Koscielny S, Rezvani A, Laplanche A, Estève J, Ferlay J and de Vathaire F (eds) (1996) *Estimation de l'incidence des cancers en France, 1983–1987*. Editions INSERM: Paris

Dietmaier W, Wallinger S, Bocker T, Kullmann F, Fishel R and Rüschoff J (1997) Diagnostic microsatellite instability: definition and correlation with mismatch repair protein expression. *Cancer Res* **57**: 4749–4756

Drummond JT, Li G, Longley MJ and Modrich P (1995) Isolation of an hMSH2-p160 heterodimer that restores DNA mismatch repair to tumor cells. *Science* **268**: 1909–1912

Fishel R, Lescoe MK, Rao MRS, Copeland NG, Jenkins NA, Garber J and Kolodner R (1993) The human mutator gene homolog MSH2 and its association with hereditary non polyposis colon cancer. *Cell* **75**: 1027–1038

Fishel R, Ewel A, Lee S, Lescoe MK and Griffith J (1994) Binding of mismatched microsatellite DNA sequences by the human MSH2 protein. *Science* **266**: 1403–1405

Fujiwara T, Stolker JM, Watanabe T, Rashid A, Longo P, Eshleman JR, Booker S, Lynch HT, Jass JR, Green JS, Kim H, Jen J, Vogelstein B and Hamilton SR (1998) Accumulated clonal genetic alterations in familial and sporadic colorectal carcinomas with widespread instability in microsatellite sequences. *Am J Pathol* **153**: 1063–1078

Futreal PA, Liu QY, Shattuckeidens D, Cochran C, Harshman K, Tavtigian S, Bennett LM, Haugenstrano A, Swensen J, Miki Y, Eddington K, McClure M, Frye C, Weaverfeldhaus J, Ding W, Gholami Z, Soderkvist P, Terry L, Jhanwar S, Berchuck A, et al. (1994) BRCA1 mutations in primary breast and ovarian carcinomas. *Science* **266**: 120–122

Genuardi M, Anti M, Capozzi E, Leonardi F, Fornasarig M, Novella E, Bellacosa A, Valenti A, Gasbarrini GB, Roncucci L, Benatti P, Percesepe A, Ponz de Leon M, Coco C, de Paoli A, Valentini M, Boiocchi M, Neri G and Viel A (1998*a*) MLH1 and MSH2 constitutional mutations in colorectal cancer families not meeting the standard criteria for hereditary nonpolyposis colorectal cancer. *Int J Cancer* **75**: 835–839

Genuardi M, Viel A, Bonora D, Capozzi E, Bellacosa A, Leonardi F, Valle R, Ventura A, Pedroni M, Boiocchi M and Neri G (1998*b*) Characterization of MLH1 and MSH2 alternative splicing and its relevance to molecular testing of colorectal cancer susceptibility. *Hum Genet* **102**: 15–20

Gompel A, Sabourin JC, Martin A, Yaneva H and Poitout P (1994) BCL-2 expression in the endometrium during the menstrual cycle. *Am J Pathol* **144**: 1195–1202

Gyapay G, Morissette J, Vignal A, Dib C, Fizames C, Millasseau P, Marc S, Bernardi G, Lathrop M and Weissenbach J (1994) The 1993–1994 Généthon human genetic linkage map. *Nature Genet* **7**: 246–339

Hemminki A, Peltomaki P, Mecklin J, Järvinen H, Salovaara R, Nyström-Lahti M, de la Chapelle A and Aaltonen LA (1994) Loss of the wild type *MLH1* gene is a feature of hereditary nonpolyposis colorectal cancer. *Nature Genet* **8**: 405–410

Herman JG, Umar A, Polyak K, Graff JR, Ahuja N, Issa JPJ, Markowitz S, Willson JKV, Hamilton SR, Kinzler KW, Kane MF, Kolodner RD, Vogelstein B, Kunkel TA and Baylin SB (1998) Incidence and functional consequences of hMLH1 promoter hypermethylation in colorectal carcinoma. *Proc Natl Acad Sci USA* **95**: 6870–6875

Ionov Y, Peinado MA, Malkhosyan S, Shibata D and Perucho M (1993) Ubiquitous somatic mutations in simple repeated sequences reveal a new mechanism for colonic carcinogenesis. *Nature* **363**: 558–561

Janin N (2000) A simple model for carcinogenesis of colorectal cancers with microsatellite instability. *Adv Cancer Res* **77**: 189–221

Kane MF, Loda M, Gaida GM, Lipman J, Mishra R, Goldman H, Jessup JM and Kolodner R (1997) Methylation of the hMLH1 promoter correlates with lack of expression of hMLH1 in sporadic colon tumors and mismatch repair-defective human tumor cell line. *Cancer Res* **57**: 808–811

Kinzler K and Vogelstein B (1996) Lessons from hereditary colorectal cancer. *Cell* **87**: 159–170

Kinzler KW and Vogelstein B (1997) Gatekeepers and caretakers. *Nature* **386**: 761–763

Kohonen-Corish M, Ross VL, Doe WF, Kool DA, Edkins E, Faragher I, Wijnen J, Meera Khan P, Macrae F and St John DJB (1996) RNA-based mutation screening in hereditary nonpolyposis colorectal cancer. *Am J Hum Genet* **59**: 818–824

Konishi M, Kikuchi-Yanoshita R, Tanaka K, Muraoka M, Onda A, Okumura Y, Kishi N, Iwama T, Mori T, Koike M, Ushio K, Chiba M, Nomizu S, Konishi F, Utsunomiya J and Miyaki M (1996) Molecular nature of colon tumors in hereditary nonpolyposis colon cancer, familial polyposis, and sporadic colon cancer. *Gastroenterology* **111**: 307–317

Kowalski LD, Mutch DG, Herzog TJ, Rader JS and Goodfellow PJ (1997) Mutational analysis of *MLH1* and *MSH2* in 25 prospectively-acquired RER+ endometrial cancers. *Genes Chromosomes Cancer* **18**: 219–227

Leach FS, Nicolaides NC, Papadopoulos N, Liu B, Jen J, Parsons R, Peltomaki P, Sistonen P, Aaltonen LA, Nystromlahti M, Guan XY, Zhang J, Meltzer PS, Yu JW, Kao FT, Chen DJ, Cerosaletti KM, Fournier REK, Todd S, Lewis T, et al. (1993) Mutations of a mutS homolog in hereditary nonpolyposis colorectal cancer. *Cell* **75**: 1215–1225

Liu B, Farrington SM, Petersen GM, Hamilton SR, Parsons R, Papadopoulos N, Fujiwara T, Jen J, Kinzler KW, Wyllie AH, Vogelstein B and Dunlop MG (1995*a*) Genetic instability occurs in the majority of young patients with colorectal cancer. *Nat Med* **1**: 348–352

Liu B, Nicolaides NC, Markowitz S, Willson JKV, Parsons RE, Jen J, Papadopoulos N, Peltomäki P, de la Chapelle A, Hamilton SR, Kinzler KW and Vogelstein B (1995*b*) Mismatch repair gene defects in sporadic colorectal cancers with microsatellite instability. *Nat Genet* **9**: 48–55

Liu B, Parsons R, Papadopoulos N, Nicolaides NC, Lynch HT, Watson P, Jass JR, Dunlop M, Wyllie A, Peltomaki P, de la Chapelle A, Hamilton SR, Vogelstein B and Kinzler KW (1996) Analysis of mismatch repair genes in hereditary non-polyposis colorectal cancer patients. *Nat Med* **2**: 169–174

Losi L, Ponz de Leon M, Jiricny J, Di Gregorio C, Benatti P, Percesepe A, Fante R, Roncucci L, Pedroni M and Benhattar J (1997) K-ras and p53 mutations in hereditary non-polyposis colorectal cancers. *Int J Cancer* **74**: 94–96

Lu S, Kawabata M, Imamura T, Akiyama Y, Nomizu T, Miyazono K and Yuasa Y (1998) HNPCC associated with germline mutation in the TGFβ type II receptor gene. *Nat Genet* **19**: 17–18

Lynch HT and Smyrk T (1996) Hereditary non-polyposis colorectal cancer (Lynch syndrome). An updated review. *Cancer* **78**: 1149–1167

Lynch HT, Ens J, Lynch JF and Watson P (1988) Tumor variation in three extended Lynch syndrome II kindreds. *Am J Gastroenterol* **83**: 741–747

Lynch HT, Smyrk T and Lynch JF (1996) Overview of natural history, pathology, molecular genetics and management of HNPCC (Lynch syndrome). *Int J Cancer (Pred Oncol)* **69**: 38–43

Merajver SD, Pham TM, Caduff RF, Chen M, Poy EL, Cooney KA, Weber BL, Collins FS, Johnston C and Franck TS (1995) Somatic mutations in the *BRCA1* gene in sporadic ovarian tumours. *Nature Genet* **9**: 439–443

Midgley CA and Lane DP (1997) p53 protein stability in tumour cells is not determined by mutation but is dependent on Mdm2 binding. *Oncogene* **15**: 1179–1189

Miyaki M, Konishi M, Tanaka K, Kikuchi-Yanoshita R, Muraoka M, Yasuno M, Igari T, Koike M, Chiba M and Mori T (1997) Germline mutation of *MSH6* as the cause of hereditary nonpolyposis colorectal cancer. *Nat Genet* **17**: 271–272

Mori Y, Shiwaku H, Fukushige S, Wakatsuki S, Sato M, Nukiwa T and Horii A (1997) Alternative splicing of *hMSH2* in normal human tissues. *Hum Genet* **99**: 590–595

Moslein G, Tester DJ, Lindor NM, Honchel R, Cunningham JM, French AJ, Halling KC, Schwab M, Goretzki P and Thibodeau SN (1996) Microsatellite instability and mutation analysis of *hMSH2* and *hMLH1* in patients with sporadic, familial and hereditary colorectal cancer. *Hum Mol Genet* **5**: 1245–1252

Munemitsu S, Albert I, Souza B, Rubinfeld B and Polakis P (1995) Regulation of intracellular beta-catenin levels by the adenomatous polyposis coli (APC) tumor-suppressor protein. *Proc Natl Acad Sci USA* **92**: 3046–3050

Nicolaides NC, Papadopoulos N, Liu B, Wei Y, Carter KC, Ruben SM, Rosen CA, Haseltine WA, Fleischmann RD, Fraser CM, Adams MD, Venter JC, Dunlop MG, Hamilton SR, Petersen GM, de la Chapelle A, Vogelstein B and Kinzler KW (1994) Mutations of two *PMS* homologs in hereditary non polyposis colon cancer. *Nature* **371**: 75–80

Nicolaides NC, Littman SJ, Modrich P, Kinzler KW and Vogelstein B (1998) A naturally occurring hPMS2 mutation can confer a dominant negative mutator phenotype. *Mol Cell Biol* **18**: 1635–1641

*© 2000 Cancer Research Campaign*

Olschwang S, Hamelin R, Laurent-Puig P, Thuille B, De Rycke Y, Li Y, Muzeau F, Girodet J, Salmon R and Thomas G (1997) Alternative genetic pathways in colorectal carcinogenesis. *Proc Natl Acad Sci USA* **94**: 12122–12123

Orth K, Hung J, Gazdar A, Bowcock A, Mathis M and Sambrook J (1994) Genetic instability in human ovarian cancer cell lines. *Proc Natl Acad Sci USA* **91**: 9495–9499

Papadopoulos N, Nicolaides N, Wei Y, Ruben SM, Carter KC, Rosen CA, Haseltine WA, Fleischmann RD, Fraser CM, Adams MD, Venter JC, Hamilton SR, Petersen GM, Watson P, Lynch HT, Peltomäki P, Mecklin J, de la Chapelle A, Kinzler K and Vogelstein B (1994) Mutation of a *mutL* homolog in hereditary colon cancer. *Science* **263**: 1625–1629

Parsons R, Myeroff LL, Liu B, Willson JKV, Markowitz SD, Kinzler KW and Vogelstein B (1995) Microsatellite instability and mutations of the transforming growth factor β type II receptor gene in colorectal cancer. *Cancer Res* **55**: 5548–5550

Prolla TA, Pang Q, Alani E, Kolodner RD and Liskay RM (1994) MLH1, PMS1, and MSH2 interactions during the initiation of DNA mismatch repair in yeast. *Science* **265**: 1091–1093

Richards B, Zhang H, Phear G and Meuth M (1997) Conditional mutator phenotypes in hMSH2-deficient tumor cell lines. *Science* **277**: 1523–1526

Rodriguez-Bigas MA, Boland CR, Hamilton SR, Henson DE, Jass JR, Khan PM, Lynch H, Perucho M, Smyrk T, Sobin L and Srivastava S (1997) A National Cancer Institute workshop on Hereditary Non-polyposis Colorectal Cancer: meeting highlights and Bethesda guidelines. *J Natl Cancer Inst* **89**: 1758–1762

Shimodaira H, Filosi N, Shibata H, Suzuki T, Radice P, Kanamaru R, Friend SH, Kolodner RD and Ishioka C (1998) Functional analysis of human *MLH1* mutations in *Saccharomyces cerevisiae*. *Nature Genetics* **19**: 384–389

Soufir N, Avril MF, Chompret A, Demenais F, Bombled J, Spatz A, Stoppa-Lyonnet D, the French Familial Melanoma Study Group, Bénard J and Bressac-de Paillerets B (1998) Prevalence of p16 and CDK4 germline mutations in 48 melanoma-prone families in France. *Hum Mol Genet* **7**: 209–216

Thibodeau SN, French AJ, Roche PC, Cunningham JM, Tester DJ, Lindor NM, Moslein G, Baker SM, Liskay RM, Burgart LJ, Honchel R and Halling KC (1996) Altered expression of hMSH2 and hMLH1 in tumors with microsatellite instability and genetic alterations in mismatch repair genes. *Cancer Res* **56**: 4836–4840

Tomlinson IP, Beck NE, Homfray T, Harocopos CJ and Bodmer WF (1997) Germline HNPCC gene variants have little influence on the risk for sporadic colorectal cancer. *J Med Genet* **34**: 39–42

Vasen HFA, Offerhaus GJA, den Hartog Jager FCA, Menko FH, Nagengast FM, Griffioen G, van Hogezand RB and Heintz APM (1990) The tumour spectrum in hereditary non-polyposis colorectal cancer: a study of 24 kindreds in the Netherlands. *Int J Cancer* **46**: 31–34

Vasen HFA, Mecklin J, Meera Khan P and Lynch HT (1991) The International Collaborative Group on Hereditary Non-Polyposis Colorectal Cancer (ICG-HNPCC). *Dis Colon Rectum* **34**: 424–425

Vasen HFA, Wijnen JT, Menko FH, Kleibeuker JH, Taal BG, Griffioen G, Nagengast FM, Meijers-Heijboer EH, Bertario L, Varesco L, Bisgaard M, Mohr J, Fodde R and Meera Khan P (1996) Cancer risk in families with hereditary nonpolyposis colorectal cancer diagnosed by mutation analysis. *Gastroenterology* **110**: 1020–1027

Wang Q, Desseigne F, Lasset C, Saurin J, Navarro C, Yagci T, Keser I, Bagci H, Luleci G, Gelen T, Chayvialle J, Puisieux A and Ozturk M (1997) Germline *hMSH2* and *hMLH1* gene mutations in incomplete HNPCC families. *Int J Cancer* **73**: 831–836

Watson P and Lynch HT (1993) Extracolonic cancer in hereditary non-polyposis colorectal cancer. *Cancer* **71**: 677–685

Weber T (1996) Clinical surveillance recommendations adopted for HNPCC. *Lancet* **348**: 465

Xia L, Shen W, Ritacca F, Mitri A, Madlensky L, Berk T, Cohen Z, Gallinger S and Bapat B (1996) A truncated *hMSH2* transcript occurs as a common variant in the population: implication for genetic diagnosis. *Cancer Res* **56**: 2289–2292

 © 2000 Cancer Research Campaign
